# CO_2_/HCO_3_^−^ Accelerates Iron Reduction through Phenolic Compounds

**DOI:** 10.1128/mBio.00085-20

**Published:** 2020-03-10

**Authors:** Felix Müller, Johanna Rapp, Anna-Lena Hacker, André Feith, Ralf Takors, Bastian Blombach

**Affiliations:** a Institute of Biochemical Engineering, University of Stuttgart, Stuttgart, Germany; b Microbial Biotechnology, Campus Straubing for Biotechnology and Sustainability, Technical University of Munich, Straubing, Germany; Korea Advanced Institute of Science and Technology

**Keywords:** iron homeostasis, iron reduction, carbon dioxide, bicarbonate, DtxR, pathogens, *Corynebacterium glutamicum*, iron homeostasis

## Abstract

Iron is a vital mineral for almost all living organisms and has a pivotal role in central metabolism. Despite its great abundance on earth, the accessibility for microorganisms is often limited, because poorly soluble ferric iron (Fe^3+^) is the predominant oxidation state in an aerobic environment. Hence, the reduction of Fe^3+^ is of essential importance to meet the cellular demand of ferrous iron (Fe^2+^) but might become detrimental as excessive amounts of intracellular Fe^2+^ tend to undergo the cytotoxic Fenton reaction in the presence of hydrogen peroxide. We demonstrate that the complex formation rate of Fe^3+^ and phenolic compounds like protocatechuic acid was increased by 46% in the presence of HCO_3_^−^ and thus accelerated the subsequent redox reaction, yielding reduced Fe^2+^. Consequently, elevated CO_2_/HCO_3_^−^ levels increased the intracellular Fe^2+^ availability, which resulted in at least 50% higher biomass-specific fluorescence of a DtxR-based Corynebacterium glutamicum reporter strain, and stimulated growth. Since the increased Fe^2+^ availability was attributed to the interaction of HCO_3_^−^ and chemical iron reduction, the abiotic effect postulated in this study is of general relevance in geochemical and biological environments.

## Abstract (precis)

In an oxygenic environment, poorly soluble Fe^3+^ must be reduced to meet the cellular Fe^2+^ demand. This study demonstrates that elevated CO_2_/HCO_3_^−^ levels accelerate chemical Fe^3+^ reduction through phenolic compounds, thus increasing intracellular Fe^2+^ availability. A number of biological environments are characterized by the presence of phenolic compounds and elevated HCO_3_^−^ levels and include soil habitats and the human body. Fe^2+^ availability is of particular interest in the latter, as it controls the infectiousness of pathogens. Since the effect postulated here is abiotic, it generally affects the Fe^2+^ distribution in nature.

## INTRODUCTION

Iron is a vital mineral for almost all living organisms and participates in inevitable electron transfer reactions since it serves a redox potential from −500 to +300 mV by switching between two oxidation states (Fe^2+^/Fe^3+^) (1). Despite its great abundance in the Earth’s crust (representing the fourth most abundant element), its availability for organisms is limited, because it is mainly present as poorly soluble Fe^3+^ (10^−18^ M at pH 7.0) in an oxidative environment (2). Sophisticated strategies to increase its accessibility have evolved, including the secretion of siderophores and smaller iron chelators that enhance the solubility of Fe^3+^ (3). Iron complexes are then taken up via designated transport systems in an energy-dependent fashion (2). Since reduced Fe^2+^ is incorporated as a prosthetic group in a number of enzymes that belong to respiratory complexes or that participate in tricarboxylic acid (TCA) cycle reactions and stress response inside the cell, the reduction of Fe^3+^ is of central relevance. Several studies on the characterization of ferric reductase activity are reviewed by Schröder et al. (4). It turned out that most designated ferric reductases are in fact flavin reductases that regenerate the substrate for chemical iron reduction. This is not limited to intracellular reactions, since it was shown recently that the human pathogen Listeria monocytogenes possesses an extracellular electron transfer apparatus, in which iron reduction is mediated by flavin (5).

In the Gram-positive soil bacterium Corynebacterium glutamicum, which is used in amino acid production on an industrial scale, little is known about iron uptake mechanisms because most annotated genes lack experimental validation (6, 7). During the development of a suitable minimal medium for C. glutamicum, the addition of citrate (8) or small amounts of the diphenolic compounds catechol or protocatechuic acid (PCA) (3,4-dihydroxybenzoic acid) revealed a growth-promoting effect, which was attributed to the improved iron uptake of Fe^3+^ chelates (9). To ensure sufficient initial iron uptake, PCA became a component of the widely used CgXII medium (10).

Excessive amounts of intracellular iron are deposited in specialized storage proteins (ferritin [Ftn] and Dps) and can be remobilized from there in times of iron starvation (2). A novel mode of iron remobilization was identified recently in C. glutamicum. Pupylation of a surface lysine residue of the Ftn and Dps proteins initiates the unfolding and subsequent Fe^3+^ release in the cytosol. However, the mechanism responsible for reduction of Fe^3+^ remains unclear (11).

Diphenolic substances (e.g., 2,3-dihydroxybenzoic acid [2,3-DHB] and derivatives) are secreted under iron-restricted growth conditions by a variety of organisms such as Bacillus subtilis and *Paracoccus* (*Micrococcus*) *denitrificans* (3, 12, 13). It was suggested that 2,3-DHB and derivatives are involved in iron uptake, as mutants deficient in the biosynthesis of 2,3-DHB and derivatives imported less iron, and adding these compounds enhanced iron transport (14). PCA is a structural relative of the common catecholate-type siderophore precursor 2,3-DHB and represents the basic compound of the rather unusual siderophore petrobactin (15). Bacillus anthracis and Bacillus cereus secrete great amounts of PCA in response to iron limitation (16–18). As a diphenolic Fe^3+^ chelator (19), it was expected to serve a function analogous to 2,3-DHB enhancing iron uptake, although evidence for this had not been provided (16).

Besides chelating iron, diphenols have the potential to reduce Fe^3+^ (20). The redox reaction in Fe^3+^-PCA chelates (19, 21)—and generally speaking in Fe^3+^-monocatecholate complexes—proceeds via the sequential oxidation of two adjacent hydroxyl groups to the respective semiquinone and quinone, providing two electrons per molecule for the reduction of Fe^3+^ (20). The reaction depends on an acidic pH, as iron and PCA bind in an 1:1 stoichiometry at a pH of <4.5 (19), and the redox reaction is inhibited by the presence of (at least) the biscatecholate complex (20, 21).

The phenotypical response of bacteria to different CO_2_/HCO_3_^−^ levels has been extensively reviewed with a biotechnological, pathogenic, or environmental focus (22–25). On the one hand, the effect on cell viability might be detrimental, considering that CO_2_-based sterilization operating at high pressure is used in food processing. However, on the other hand, a class of bacteria called capnophiles demonstrates that HCO_3_^−^ can be a vital substrate as they are unable to grow unless sufficiently high CO_2_ levels are established (26). Carboxylation reactions might generally benefit from the increased availability of CO_2_/HCO_3_^−^. C. glutamicum is especially well equipped with enzymes catalyzing these reactions, e.g., at the phosphoenolpyruvate (PEP)-pyruvate-oxaloacetate node (27). As a consequence, higher product (e.g., succinate) and biomass yields per substrate were reported in C. glutamicum at elevated CO_2_ levels and could be attributed particularly to an increased flux via the anaplerotic reactions (28–30). Anaerobic growth on glucose and tryptone could be enhanced by increasing concentrations of CO_2_. The authors suggest that this might enhance acetyl coenzyme A (acetyl-CoA) carboxylation reactions, yielding greater levels of fatty and mycolic acids (31). Although the exponential growth rate of C. glutamicum could not be promoted under aerobic conditions, elevated CO_2_ in the inlet air of a bioreactor cultivation provoked the transcriptional response of almost the entire DtxR regulon (30). The master regulator of iron homeostasis in C. glutamicum was originally named after its functional homologue in the pathogenic C. diphtheriae, where it controls the toxin production in response to the iron availability (32). In fact, to date, many pathogens that induce the expression of toxin genes under iron starvation conditions and typically integrate this signal via the transcriptional regulators Fur, DtxR and homologues thereof are known (33–35). In analogy with the iron limitation, elevated CO_2_/HCO_3_^−^ levels represent another suitable indicator for the host environment (22). The expression of toxin-encoding genes in Vibrio cholerae or Pseudomonas aeruginosa is mediated by the transcriptional regulators ToxT and RegA in response to elevated HCO_3_^−^ concentrations (23). Interestingly, *regA* expression in P. aeruginosa is in turn repressed in an iron-dependent manner by a Fur homologue (33). Hence, understanding the interaction between CO_2_/HCO_3_^−^ and iron availability is particularly interesting with regard to toxin production by pathogenic bacteria (23).

## RESULTS

### Elevated levels of CO_2_/HCO_3_^−^ increase intracellular Fe^2+^ availability.

To monitor intracellular Fe^2+^ availability, we constructed the fluorescence-based reporter strain C. glutamicum FEM3 which responds to the activation state of DtxR (see Fig. S1 in the supplemental material). Therefore, we chromosomally integrated the *lacI* gene under the control of the *ripA* promoter (P*_ripA_*), which is controlled by DtxR. High Fe^2+^ concentrations induce DtxR binding to P*_ripA_*, thus provoking repression of *lacI* and ultimately resulting in *egfp* expression under the control of the strong *tac* promoter (P*_tac_*). Thus, the genetic circuit of C. glutamicum FEM3 constitutes a signal amplifier and a converter of the native repression mechanism.

10.1128/mBio.00085-20.1 FIG S1 Schematic overview of enhanced green fluorescent protein (eGFP) production in the DtxR-based reporter strain C. glutamicum FEM3. Activation of DtxR (by Fe^2+^ binding) causes repression of *lacI* expression via binding of DtxR to the operator in the promoter of *ripA*. This event results in expression of *egfp* under the control of the strong *tac* promoter. Download FIG S1, EPS file, 0.2 MB. Copyright © 2020 Müller et al. 2020 Müller et al. This content is distributed under the terms of the Creative Commons Attribution 4.0 International license.

Under all conditions tested, C. glutamicum FEM3 and the wild type (WT) showed identical growth. Cultivating C. glutamicum FEM3 under iron starvation (1 μM) resulted in a marginal increase of the fluorescence per biomass [78 ± 4 arbitrary units (a.u.) · (g_CDW_ liter^−1^)^−1^ where g_CDW_ is the cell weight (dry weight) in grams] over the autofluorescence of the WT [64 ± 3 a.u. · (g_CDW_ liter^−1^)^−1^] after 24 h. At iron excess (100 μM), C. glutamicum FEM3 yielded a fluorescence per biomass which was about fourfold higher [313 ± 20 a.u. · (g_CDW_ liter^−1^)^−1^] at the end of the cultivation compared to conditions under iron starvation (Fig. S2).

10.1128/mBio.00085-20.2 FIG S2 Validation of autofluorescence (WT C. glutamicum) and fluorescence of reporter strain C. glutamicum FEM3 in response to iron starvation (1 μM FeSO_4_) and excess (100 μM FeSO_4_). Fluorescence was normalized with regard to the biomass concentration after 24-h cultivation. Bars represent mean values, and error bars indicate standard deviations for three independent biological replicates. Download FIG S2, EPS file, 0.2 MB. Copyright © 2020 Müller et al. 2020 Müller et al. This content is distributed under the terms of the Creative Commons Attribution 4.0 International license.

**FIG 1:**
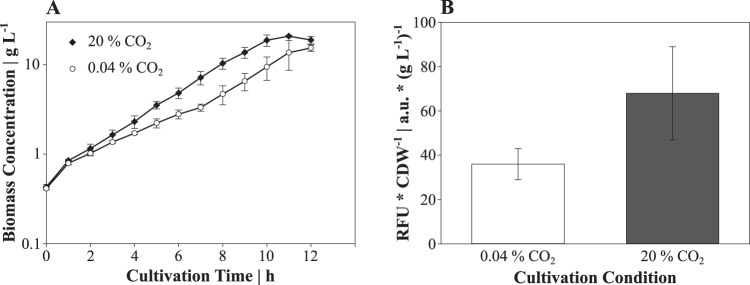
Biomass formation of C. glutamicum FEM3 (A) and biomass-specific fluorescence after 24 h of cultivation in a parallel bioreactor setup (B). C. glutamicum FEM3 was cultivated aerobically in minimal medium with 20 g glucose liter^−1^ with synthetic air containing 20% CO_2_ (21% O_2_, 59% N_2_) or ambient air (0.04% CO_2_). Data points (A) and bars (B) represent mean values, and error bars indicate standard deviations for three biological independent replicates.

**FIG 2:**
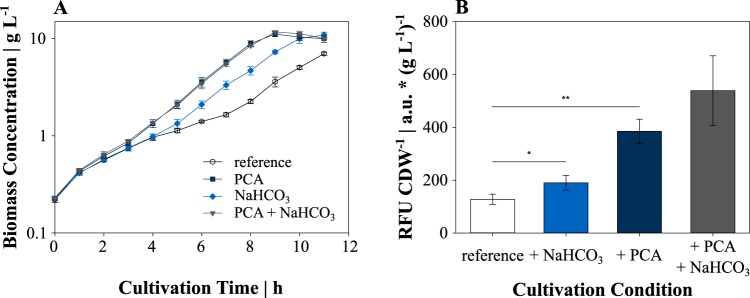
(A) Shaking flask cultivations of wild-type (WT) C. glutamicum in minimal medium with 20 g glucose liter^−1^ without supplement (reference), with 195 μM PCA, 50 mM NaHCO_3_ or a combination of both supplements. (B) Biomass-specific fluorescence of C. glutamicum FEM3 after 24 h of cultivation with the indicated supplements. Data points and bars represent mean values with error bars indicating standard deviations for 3 to 10 independent biological replicates. Values that are significantly different by a two-sample *t* test are indicated by bars and asterisks as follows: *, *P* < 0.05; **, *P* < 0.01.

Having proven the functionality at different iron concentrations, we cultivated C. glutamicum FEM3 aerobically in a parallel bioreactor system to evaluate the intracellular Fe^2+^ availability in response to altering CO_2_/HCO_3_^−^ levels. With 20% CO_2_ in the inlet air, C. glutamicum FEM3 grew exponentially (growth rate [μ] = 0.36 ± 0.02 h^−1^) until the carbon source glucose was exhausted (Fig. 1A). In contrast, aeration with an ambient CO_2_ concentration (0.04%) provoked biphasic growth of C. glutamicum FEM3 with a growth rate of 0.26 ± 0.05 h^−1^ and 0.32 ± 0.02 h^−1^ in growth phase one and two, respectively (Fig. 1A). Biomass-specific fluorescence started at similar levels in both conditions but after 24 h on average reached almost twofold-higher values at 20% CO_2_ content, whereas it remained at a low level at the ambient CO_2_ concentration (Fig. 1B). Biphasic growth of C. glutamicum FEM3 and the WT was also observed on minimal medium containing glucose in shaking flasks at an ambient CO_2_ concentration (Fig. 2A). In accordance with the bioreactor cultivations, growth was stimulated by adding 30 mM NaHCO_3_, which shortened the initial (nonexponential) growth phase from about 7 h under reference conditions to 4 h and led to a growth rate of 0.40 ± 0.03 h^−1^ during the second growth phase. The addition of 195 μM of the iron chelator PCA restored exponential growth throughout the cultivation (Fig. 2A) and resulted in a slightly higher growth rate compared to the rate for the second growth phase of the reference condition (reference, μ = 0.37 ± 0.01 h^−1^; PCA, μ = 0.43 ± 0.01 h^−1^).

The biomass-specific fluorescence of the shaking flask approach showed a 0.5- and 3.0-fold increase in response to the supplementation of NaHCO_3_ or PCA compared to the reference cultivation after 24 h, respectively (Fig. 2B). Although growth of C. glutamicum FEM3 was not further improved by the combined addition of NaHCO_3_ and PCA (Fig. 2A), there might be a synergistic effect on fluorescence level (4.2-fold compared to the reference; Fig. 2B), which was, however, not significantly higher than in the PCA-supplemented condition (by Student’s *t* test). Supplementing with PCA or NaHCO_3_ did not show a growth-stimulating effect in iron-depleted medium (Fig. S3), where biomass stagnated at a final 0.9 to 1.1 g liter^−1^ regardless of supplementation. In summary, these results demonstrate that in the presence of the extracellular iron source (FeSO_4_), elevated levels of CO_2_/HCO_3_^−^ and/or the addition of PCA to the minimal medium results in an increased intracellular Fe^2+^ availability in C. glutamicum.

10.1128/mBio.00085-20.3 FIG S3 Biomass generation of WT C. glutamicum in iron-depleted (0 μM FeSO_4_; 250 μM 2,2'-dipyridyl) CgXII minimal medium containing 20 g glucose liter^−1^ and supplemented with PCA (195 μM) or NaHCO_3_ (30 mM) or without additives (reference). Data points of duplicate experiments and triplicate experiments (after 0 and 5 h) are shown individually. Download FIG S3, EPS file, 0.2 MB. Copyright © 2020 Müller et al. 2020 Müller et al. This content is distributed under the terms of the Creative Commons Attribution 4.0 International license.

### CO_2_*/*HCO_3_^−^ does not increase thermal stability of the DtxR protein or interact with iron storage and mobilization.

The DtxR protein of C. glutamicum shares 72% sequence identity with its extensively characterized homologue from Corynebacterium diphtheriae, including conserved ligand binding sites. Dimerization of the latter and subsequent DNA recognition are induced by sequential binding of two divalent metal ions per monomer. Besides the physiological effector of DtxR, Fe^2+^, *in vitro* activation was demonstrated with Ni^2+^, Mn^2+^, and Co^2+^ at various affinities (36). As they are stable in an oxidative environment, they are commonly found in crystallographic structures of DtxR. An anion binding site is located in close proximity to the low-affinity binding site and essential for coordination of the metal ion (37–39). To address the question whether binding of HCO_3_^−^ to the anion binding site of DtxR could cause the higher activation, we performed differential scanning fluorimetry. The thermal stability of purified DtxR protein was enhanced by increasing concentrations of divalent metal ions (Ni^2+^ and Mn^2+^) and dissociation constants (*K_D_*) were calculated to 5.3 ± 1.1 μM and 21.8 ± 3.6 μM, respectively. However, addition of up to 100 mM NaHCO_3_ did not stabilize DtxR further at any concentration of metal ions (data not shown).

To investigate whether elevated CO_2_/HCO_3_^−^ levels have an impact on iron storage or mobilization, we created deletion mutations (Δ*ftn*, Δ*dps*, Δ*ftn* Δ*dps*, and Δ*pup*) in C. glutamicum FEM3 and the WT. The deletion mutants lack either one (Δ*ftn*, Δ*dps*) or both iron storage proteins (Δ*ftn* Δ*dps*) or are unable to initiate remobilization of the stored iron by pupylation (Δ*pup*). With the exception of C. glutamicum Δ*pup*, none of the deletion mutants showed a difference regarding growth and fluorescence in any of the conditions tested (data not shown). C. glutamicum Δ*pup* revealed an even stronger growth defect than the WT under reference conditions. However, supplementation of PCA or NaHCO_3_ restored the respective growth phenotype with the result that C. glutamicum Δ*pup* and the WT grew identically again (Fig. S4).

10.1128/mBio.00085-20.4 FIG S4 Biomass generation of C. glutamicum WT and C. glutamicum Δ*pup* during shaking flask cultivation in glucose-containing minimal medium supplemented with PCA (195 μM) or NaHCO_3_ (30 mM) or without additives (reference). Data points represent mean values, and error bars indicate standard deviations for three independent biological replicates. Download FIG S4, EPS file, 0.2 MB. Copyright © 2020 Müller et al. 2020 Müller et al. This content is distributed under the terms of the Creative Commons Attribution 4.0 International license.

### CO_2_*/*HCO_3_^−^ accelerates Fe^3+^ reduction through phenolic compounds.

**FIG 3:**
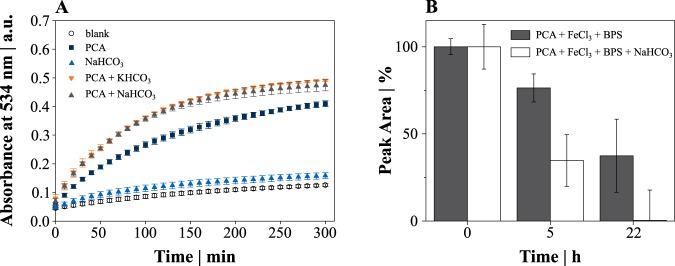
(A) Kinetic analysis of the Fe^2+^-BPS complex formation at 534 nm with 19.5 μM PCA and/or 50 mM HCO_3_^−^. (B) Relative PCA degradation over time in the presence and absence of 50 mM NaHCO_3_. Data points and bars represent mean values with error bars indicating standard deviations for three to six independent replicates.

Since we could not attribute the improved Fe^3+^ reduction capacity in the presence of elevated CO_2_/HCO_3_^−^ levels to an interaction with stored intracellular iron, we focused on the chemical reduction by PCA and HCO_3_^−^ using the Fe^2+^-specific iron chelator bathophenanthroline disulfonic acid (BPS) for detection. After 5 h of incubation, the amount of Fe^2+^-BPS complexes was about sevenfold higher in the presence of 19.5 μM PCA compared to a sample without additives (blank control; Fig. 3A). Although the addition of 50 mM NaHCO_3_ alone increased the final absorbance only marginally, it promoted the iron reduction in the presence of PCA significantly. Differences caused by the presence of NaHCO_3_ were most remarkable during the initial reduction phase with a >50% greater amount of Fe^2+^-BPS complexes up to 100 min after the start of the assay (Fig. 3A). The sample pH was maintained at 7.4 throughout the incubation in 200 mM 3-(*N*-morpholino)propanesulfonic acid (MOPS) buffer, thus proving that a shift in pH was not causing the increase in absorbance. We also replaced NaHCO_3_ with KHCO_3_, which led to the identical improvement in iron reduction (Fig. 3A). An equimolar NaCl concentration did not trigger iron reduction (data not shown).

We monitored the degradation of PCA by liquid chromatography-quadrupole time of flight mass spectrometry (LC-MS-QTOF) analysis and found only about half of the residual amount of PCA when samples were incubated for 5 h in the presence of HCO_3_^−^ compared to samples incubated without HCO_3_^−^ (Fig. 3B). Furthermore, we identified oxidation products with the chemical formula C_7_H_6_O_6_ and C_7_H_8_O_6_.

Concluding that PCA serves as electron donor in the reduction of Fe^3+^, we tested other aromatic compounds at identical concentrations that could be associated with iron reduction.

**FIG 4:**
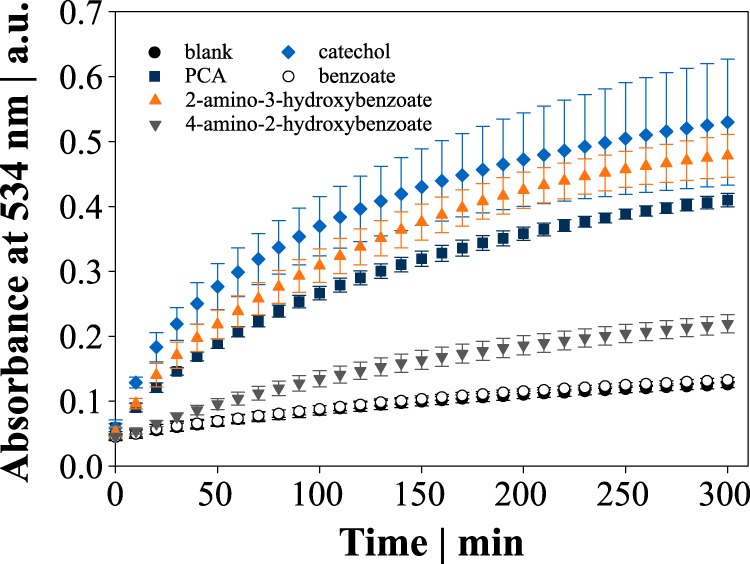
Kinetic analysis of the Fe^2+^-BPS complex formation at 534 nm with different functionalized aromatic compounds. Data points represent mean values with error bars indicating standard deviations of three to six independent replicates.

Catechol and 2-amino-3-hydroxybenzoic acid (3-hydroxyanthranilic acid [3-HAA]) carrying two adjacent hydroxyl groups or a mix of amino and hydroxyl groups, respectively, exhibited reduction capacities that were at least as high as PCA. Iron reduction by 4-amino-2-hydroxybenzoic acid (*para*-aminosalicylic acid), where the amino and hydroxyl groups are not positioned adjacent to each other, was only intermediate, whereas benzoic acid lacking hydroxyl and amino groups did not reduce iron at all (Fig. 4). The addition of 50 mM NaHCO_3_ alongside with either of the above compounds (except for benzoic acid) accelerated the iron reduction in the same way as observed with PCA.

**FIG 5:**
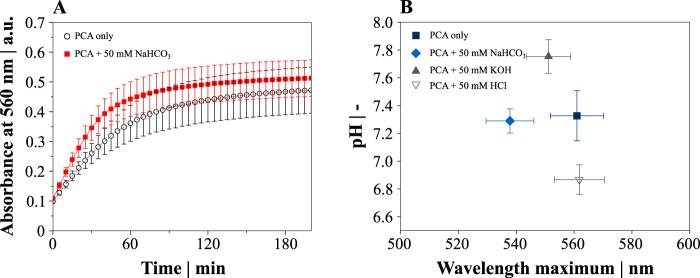
Complex formation between Fe^3+^ and PCA in the presence and absence of 50 mM NaHCO_3_. (A) The kinetics of complex formation was monitored by an increase of the absorbance at 560 nm. (B) Wavelength of the maximum absorbance (λ_max_) of the Fe^3+^-PCA complexes formed in the presence and absence of NaHCO_3_ correlated with pH. Data points represent mean values, and error bars indicate standard deviations for three to five independent replicates.

To further elucidate the role of HCO_3_^−^ in the reduction of iron, we analyzed the complex formation between Fe^3+^ and the iron chelator PCA that is required prior to reduction. In accordance with iron reduction, the initial rate of Fe^3+^-PCA complex formation (0 to 35 min) was increased by 46% through the addition of 50 mM NaHCO_3_ (5.2 ± 1.0 ΔmA_560_ min^−1^ [change in milli absorbance units at 560 nm per minute] versus 7.6 ± 1.3 ΔmA_560_ min^−1^; Fig. 5A). In addition to the altered kinetics, the maximum absorbance of those complexes shifted to a shorter wavelength (λ_max_). The assay pH was again not affected by the addition of NaHCO_3_, and intentional pH perturbation with 50 mM KOH or HCl failed to reproduce the λ_max_ shift (Fig. 5B).

## DISCUSSION

Recently, Blombach et al. (30) initially disclosed a link between a high CO_2_ proportion in the inlet air and a transcriptional response of almost the complete DtxR regulon in C. glutamicum. In this study, we demonstrate that elevated CO_2_/HCO_3_^−^ levels increased the intracellular Fe^2+^ availability and thus stimulated growth of C. glutamicum. The higher Fe^2+^ concentration was not coupled to biological activity. Instead, we identified an abiotic interaction of CO_2_/HCO_3_^−^ with chemical iron reduction through phenolic compounds.

Growth retardation had not been reported previously, when C. glutamicum cultures were aerated with pressurized air and has been introduced in this study presumably by the insertion of an additional minimal medium preculture, since all other process parameters were kept the same (30). The growth defect could be transferred to a shaking flask approach and was apparently caused by a limitation of intracellular iron availability, as suggested (i) by the lower fluorescence of FEM3, (ii) by the stronger growth retardation of C. glutamicum Δ*pup* in the reference culture, which was found to exhibit a growth retardation only in iron starvation (11) and (iii) by the fact that PCA supplementation restored exponential growth (9). Analogously, an extended lag phase of C. glutamicum in minimal medium had been observed when the main culture was inoculated from a preculture in minimal medium or when the inoculum was washed excessively and, consequently, the addition of iron chelators like catechol, PCA, or citrate to minimal medium was suggested (8, 9). Interestingly, this requirement for iron chelators became apparent only when carbonate was omitted from the medium (8, 9, 40). However, the role of CO_2_ with regard to the iron availability was not addressed further until noting a transcriptional response of the DtxR regulon (30).

The cometabolization of glucose and PCA can improve the growth rate of C. glutamicum to about 0.61 h^−1^. However, this requires essentially higher PCA concentrations and lower cell densities (40). At standard PCA concentrations (as applied in this study), growth was not found to be increased over the maximum of μ = 0.42 h^−1^ when glucose was utilized as the sole carbon and energy source (41). Thus, growth of PCA-supplemented and nonsupplemented shaking flask cultures recorded herein was in qualitative and quantitative accordance with the literature (40, 41).

If NaHCO_3_ supplementation were required for an increased flux via the anaplerotic reactions, and thus explaining the growth stimulation over a reference cultivation, the effect should persist in the simultaneous addition of PCA and NaHCO_3_, because the small amount of PCA is readily consumed and cannot provide a long-lasting surplus of necessary precursors. However, supplementing with both PCA and NaHCO_3_ did not result in a further growth improvement, and we found instead that the growth- stimulating effect of PCA and NaHCO_3_ was related to the presence of the extracellular iron source. In iron-depleted medium, growth ceased after two to three cell divisions as reported previously (42).

Upon dissolution of the iron source FeSO_4_ in CgXII medium, iron is present as reduced Fe^2+^. However, it will be quickly oxidized under aerobic conditions, yielding 99% of the initial iron concentration as Fe^3+^ after 15 min (43). Considering the standard preparation times of a shaking flask experiment and estimating the initially dissolved oxygen concentration at 7.5 to 8 mg liter^−1^ (22), the entire amount of iron provided in the medium will be oxidized before inoculation. This highlights the need of enhanced iron reduction mechanisms to obtain increased intracellular Fe^2+^ levels. Since the increased Fe^2+^ availability in the presence of elevated CO_2_/HCO_3_^−^ levels could not be attributed to a biological function, we analyzed the chemical reduction capacity of PCA and HCO_3_^−^. Results of iron reduction assays and the LC-MS-QTOF approach show that PCA served as an electron donor for the redox reaction. Having performed the experiments at physiological pH (7.4), our results are in contrast with the common understanding that these redox reactions are restricted to acidic pH values (19–21). At pH 7.4, PCA coordination with Fe^3+^ represents a mix of 2:1 and 3:1 stoichiometry (20) and was expected to inhibit redox reactions of the iron monocatecholate complexes, which are favored at pH < 4.5 (21). Furthermore, our results indicate that the primary oxidation products of PCA (quinone and semiquinone) are further degraded. The chemical formula C_7_H_6_O_6_ and C_7_H_8_O_6_ obtained by the LC-MS-QTOF approach might represent β-carboxy-(*cis-cis*)-muconic acid or β-carboxy-γ-carboxymethyl-Δ^α,β^-butenolide and butene-1,2,3-tricarboxy acid that were postulated to be degradation products, when PCA was oxidized with peroxyacetic acid (44). Interestingly, these products also form part of the enzymatic degradation of PCA via the β-ketoadipate pathway (45).

Beyond PCA, other phenolic compounds can promote bacterial growth and have been associated with iron reduction and transport in the past. Examples are the reductive mobilization of ferritin iron through PCA and derivatives (46) as well as 2-amino-3-hydroxybenzoic acid secretion by Cryptococcus neoformans which mediated the iron reduction required for Fe^2+^ uptake (47). Supplementing with catechol stimulated growth of C. glutamicum comparably to PCA (9), and the chemical reduction of Fe^3+^ through catechol oxidation is well-known and has been reviewed (20). Our results provide evidence that the postulated iron reduction is not unique to PCA but characteristic of other functionalized phenolic compounds. Reduction assays with benzoic acid and 4-amino-2-hydroxybenzoic acid suggest that the carboxyl group was not required for iron reduction and that the stabilization of the semiquinone in adjacent hydroxyl (or mixed functional) groups was advantageous for the reduction of iron (drastically reduced in 4-amino-2-hydroxybenzoic acid).

The beneficial effect of HCO_3_^−^ on the reduction of iron has not been postulated before. Remarkably, the iron reduction capacity of all aromatic compounds was enhanced in the presence of HCO_3_^−^, although iron reduction in the presence of HCO_3_^−^ alone was negligible. The promoting effect of HCO_3_^−^ on iron reduction was apparently related to the complex formation between Fe^3+^ and chelates, as exemplarily shown for PCA. Since we could not clarify the precise mechanism of interaction, one could assume that H^+^ and OH^−^ concentrations were the reason for the λ_max_ shift in analogy with an overall alteration of the absorbance due to the formation of Fe(OH)-PCA complexes (19). However, we carefully performed pH buffering and control experiments in order to rule out this possibility. The sample pH was not varied in our experiments by the addition of NaHCO_3_, and intentionally changing the pH failed to reproduce a comparable λ_max_ shift. It might be further speculated that HCO_3_^−^ enhances the iron accessibility to PCA in the form of ferric bicarbonate, since the initial complex formation rate was accelerated by 46%.

Although the presence of HCO_3_^−^ alone had no effect on the reduction of iron *in vitro*, the growth-promoting effect of CO_2_/HCO_3_^−^ correlated with increased intracellular Fe^2+^ levels reported *in vivo*. We conclude that the growth-promoting effect of CO_2_/HCO_3_^−^ requires the natural production of suitable reductants by C. glutamicum. Surprisingly, PCA production in C. glutamicum does not appear to be regulated in response to iron availability, unlike other organisms (16). The expression of *qsuB* encoding the dehydroshikimate dehydratase that catalyzes the conversion of 3-dehydroshikimic acid to PCA is controlled by QsuR in C. glutamicum and provides a shunt when chorismate accumulates (48, 49). The question of major iron reductants produced by C. glutamicum must be addressed in future research.

There is continuous debate about the redox reaction in Fe^3+^-catecholate complexes and its interpretation as antioxidant or prooxidant, since contradictory observations have been published (20, 50). It must be noted that the experimental setup is essentially decisive for the outcome. Many studies emphasized the antioxidant effect of PCA and other catecholates in the past (20). Fe^2+^ generation was monitored, e.g., by lipid oxidation experiments, which in contrast to BPS detection, do not exhibit high affinity for Fe^2+^. As a consequence, observations might differ from ours. The detection of Fe^2+^ with BPS in this study might have been beneficial for two reasons. (i) Fe^2+^ and BPS form a stable complex, thus preventing Fe^2+^ from reoxidation. (ii) Capturing Fe^2+^ in the complex constantly shifts the chemical equilibrium in favor of reduction. Thus, our *in vitro* screening system might be well suited for representing a biological context, considering the high Fe^2+^ affinity of iron processing enzymes, e.g., Escherichia coli IscA (the first enzyme of the iron sulfur cluster assembly machinery, *K_D_* = 3.3 × 10^−20^ to 5 × 10^−20^ M) (51, 52). Dissociation constants between iron and human transferrin (53) have been determined in a very similar range (2.1 × 10^−21^ and 4.2 × 10^−20^ M). However, the most obvious support of these hypothetical considerations is provided by the consistency of the results of *in vitro* and *in vivo* experiments in this study.

Given the predominance of Fe^3+^ in an oxygenic environment, the abiotic acceleration of iron reduction through HCO_3_^−^ is of global importance. The two prerequisites—the presence of functional aromatic compounds on one hand and high HCO_3_^−^ concentrations on the other hand—combine in a number of habitats. Aromatic compounds are ubiquitous in cellular metabolism, and plant-derived catecholic compounds can be released, e.g., in soils during biomass degradation or reach high concentrations in human plasma shortly after ingestion (20). A number of stress hormones and neurotransmitters, including dopamine, adrenaline, and norepinephrine, contain a functional catechol group and exhibit interesting features with regard to the iron homeostasis. The stimulating role of catecholamine on growth of a number of pathogenic bacteria in iron-restricted growth media containing serum, transferrin, or lactoferrin has been reported (54–58). Pathogens possess an outstanding role within the inhabitants of the human body as they encounter a constant battle for iron, the majority of which is sequestered in host transferrin and lactoferrin (35). Several studies demonstrated that catecholamines or dietary catechols can liberate iron from transferrin and lactoferrin (55, 59, 60) and might even reduce Fe^3+^ by promoting bacterial growth (61). The greater availability of liberated Fe^3+^ and Fe^2+^ resulting from this mechanism might be further increased at the high concentrations of CO_2_ species typically found in calcareous soils, but also in the human body, where HCO_3_^−^ concentrations can reach up to 140 mM (22). Considering the HCO_3_^−^ interaction with iron reduction demonstrated in this study thus might be of particular interest in understanding pathogenicity. Of the currently most threatening human pathogens, Pseudomonas aeruginosa, Staphylococcus aureus, Klebsiella pneumoniae, Mycobacterium tuberculosis, and pathogenic Escherichia coli strains as well as several other pathogenic bacteria regulate toxin expression on the transcriptional level in response to iron availability (33, 62–64).

In summary, we show that high CO_2_/HCO_3_^−^ concentrations accelerate the chemical reduction of Fe^3+^ through various phenolic compounds. This increases the intracellular Fe^2+^ availability and stimulates growth of C. glutamicum.

## MATERIALS AND METHODS

### Genetic manipulation, cloning, and strain construction.

Standard techniques of molecular biology were applied in accordance with the literature (65). Kits for purification of plasmids and PCR products and for isolation of genomic DNA were used according to Lange et al. (66). Cloning of plasmids and construction of C. glutamicum integration and deletion mutants have been performed as described in detail previously (66). All cloned fragments were verified by Sanger sequencing (GATC, Constance, Germany).

C. glutamicum FEM3 was composed of the chromosomally integrated sensor part and a replicative plasmid carrying the reporter gene. Expression of *lacI* was placed under the control of the DtxR-regulated promoter of the *ripA* gene (P*_ripA_*), followed by the strong *rrnB* terminator (T*_rrnB_*). Chromosomal integration of the cassette in C. glutamicum was targeted to CgLP13 (between *cg3344* and *cg3345*) by two 500-bp homologous sequences (66). C. glutamicum endogenous elements (500-bp flanks, P*_ripA_*) were amplified from genomic DNA using the primer pairs 5′(cg3344)-1 plus 5′(cg3344)-2, 3′(cg3344)-1 plus 3′(cg3344)-2, and PripA-1 plus PripA-2, respectively, and exogenous elements (*lacI*, T*_rrnB_*) were amplified from plasmid pJOE7706.1 (67) with lacI-1 plus lacI-2 and TrrnB-1 plus TrrnB-2, respectively. All PCR amplification products were simultaneously inserted into BamHI- and NheI-linearized pK19*mobsacB* (68) by isothermal assembling (69). The *egfp* gene under the control of the strong hybrid promoter P*_tac_* and T*_rrnB_* was amplified from pJOE7706.1 with primer pairs Ptac-1 plus Ptac-2 and egfp-1 plus egfp-2, respectively, and inserted into XbaI- and NotI-linearized pJC4 plasmid (70) in the same way.

For markerless deletions of the genes *pup* (*cg1689*), *ftn* (*cg2782*), and *dps* (*cg3327*) in C. glutamicum, 500-bp homologous sequences flanking the target gene were amplified with the respective primer pair (see Table S1 in the supplemental material) and simultaneously inserted in BamHI- and HindIII-linearized pK19*mobsacB* as described before.

10.1128/mBio.00085-20.6 TABLE S1 Oligonucleotides used for PCR amplification and sequencing. Sequences that overlap with neighboring sequences (Gibson Assembly) are shown in blue. Restriction sites are underlined. Download Table S1, DOCX file, 0.01 MB. Copyright © 2020 Müller et al. 2020 Müller et al. This content is distributed under the terms of the Creative Commons Attribution 4.0 International license.

For purification of the DtxR regulator protein, the *dtxR* gene was amplified from the C. glutamicum chromosomal DNA via PCR using the primer pair dtxR-1 and dtxR-2 and cloned into pJOE6089.4 by restriction and ligation using the NdeI and BamHI restriction sites. Competent E. coli DH5α was transformed with the resulting plasmid pJOE6089-*dtxR* carrying a C-terminal *Strep*-tag II fused to the *dtxR* gene by electroporation (66).

### Bacterial strains and cultivation conditions.

An overview of all bacterial strains and plasmids used in this work is given in Table S2. Permanent cultures were maintained at –70°C in 30% (vol/vol) glycerol. C. glutamicum strains were cultivated at 30°C on a rotary shaker in baffled shaking flasks (500 ml filled with 50 ml medium, 120 rpm). Kanamycin was added at a working concentration of 50 μg ml^−1^ when appropriate.

10.1128/mBio.00085-20.7 TABLE S2 Overview of the strains and plasmids used in this study. Download Table S2, DOCX file, 0.02 MB. Copyright © 2020 Müller et al. 2020 Müller et al. This content is distributed under the terms of the Creative Commons Attribution 4.0 International license.

Permanent cultures were streaked on 2× yeast tryptone (2× YT) (65) agar (18 g liter^−1^) plates and grown for 2 days. Liquid cultures (5 ml of 2× YT) were inoculated from the plate, grown overnight (O/N), and used to inoculate a 50-ml 2× YT culture, which was incubated for another 6 to 8 h. An appropriate volume of the complex preculture was harvested and resuspended in 2 ml of 0.9% (wt/vol) NaCl to inoculate a CgXII preculture to a starting optical density at 600 nm (OD_600_) of 1. The main cultures were inoculated in the same way from the CgXII O/N culture and incubated for at least 25 h.

### Shaking flask cultivation.

C. glutamicum cultivations were performed in CgXII medium (pH 7.4) containing 20 g glucose liter^−1^ supplemented with protocatechuic acid (PCA) to a final concentration of 195 μM (10) or with 30 mM NaHCO_3_ or not supplemented (71) as indicated. Since slight variations occurred in the past, the exact composition of the CgXII medium used in this study is outlined in Lange et al. (66). The functionality of the reporter strain C. glutamicum FEM3 was validated under iron starvation (1 μM FeSO_4_) and iron excess (100 μM FeSO_4_) conditions in the presence of 195 μM PCA and starting from identical precultures as described before (32). Iron-depleted growth medium was prepared with a trace element solution lacking the iron source (FeSO_4_), and the ferrous iron chelator 2,2-dipyridyl was added at a final concentration of 250 μM immediately before inoculation to remove any residual iron from the medium (42). Precultures were performed in iron excess conditions (100 μM FeSO_4_, instead of the usual 59 μM FeSO_4_).

### Bioreactor cultivation.

Bioreactor cultivations were performed in batch mode in 1.5-liter stainless steel bioreactors (30) with a starting volume of 800 ml CgXII medium containing 40 g glucose liter^−1^ as the sole carbon and energy source but lacking 3-(*N*-morpholino)propanesulfonic acid (MOPS) buffer and urea. The cultivation pH was maintained at pH 7.4 by the addition of 25% ammonium hydroxide. Two parallel fermentations were always inoculated from identical precultures to a starting OD_600_ of 2. Both reactors were operated at a total pressure of 1.5 bar and contained two six-blade Rushton-type impellers. The stirrer speed started at 300 rpm and was gradually increased to maintain the dissolved oxygen concentration above 35%. Reactor 1 was aerated with 0.5 vvm (volume of gas per volume of liquid per minute) pressurized air (0.04% CO_2_), and reactor 2 was aerated with synthetically mixed gas containing 20% CO_2_, 21% O_2_, and 59% N_2_ (20% CO_2_).

### Biomass formation.

Cell density was determined by measuring the optical density of a culture at 600 nm (OD_600_). Biomass concentration (cell weight [dry weight] [CDW] in grams liter^−1^) was calculated from it using the correlation coefficient CDW = 0.21 × OD_600_ (72) specific for the spectrophotometer (Ultrospec 10 cell density meter; GE Healthcare, Little Chalfont, UK).

### Fluorescence readings.

Fluorescence (relative fluorescence units [RFU]) was detected in 100-μl samples of the pure culture using 96-well microtiter plates in the Synergy 2 device (BioTek Instruments, Bad Friedrichshall, Germany) at 37°C (excitation wavelength/bandwidth, 485/20 nm; emission wavelength/bandwidth 528/20 nm) as described before (73). The culture background (after removing cells by centrifugation, 13,300 rpm, 1 min) was subtracted from the sample value and normalized with respect to the biomass concentration (RFU CDW^−1^).

### LC-MS-QTOF.

PCA degradation was detected by a relative decrease of the respective peak area via liquid chromatography-quadrupole time of flight mass spectrometry (LC-MS-QTOF). Samples (1-ml samples) containing 0.5 mM FeCl_3_ (from a stock in 10 mM HCl) were prepared in 1.5-ml Eppendorf cups. Samples were neutralized with 50 mM NaOH, and 19.5 μM PCA, 6.5 mM BPS, and 50 mM NaHCO_3_ were added when appropriate. At the indicated time points, 200-μl samples were transferred to fresh 1.5-ml Eppendorf cups and stored frozen at –20°C until further processing. After thawing, samples or respective standards were prepared in 60% (vol/vol) acetonitrile, 0.75 mM EDTA, and 10 mM ammonium acetate buffer (adjusted to pH 9.2) and analyzed by an Agilent 1260 bio-inert high-performance liquid chromatography (HPLC) system (Agilent Technologies, Waldbronn, Germany) coupled to an Agilent 6540 accurate-mass QTOF (Agilent Technologies, Santa Clara, CA, USA). Alkaline hydrophilic interaction liquid chromatography (HILIC) (74) and QTOF-MS (75) parameters were set as previously described, and analysis was conducted in the negative mode with a fragmentor voltage of 100 V in the MS mode. The extraction of chromatograms and integration were done in MassHunter Qualitative Analysis with the “Find by Formula” algorithm. Potential oxidation products were identified via the molecular formula calculator feature based on the accurate mass, isotope abundances, and isotope spacing (B.07.00, Agilent Technologies, Santa Clara, CA, USA).

### Iron reduction assays.

The kinetics of Fe^3+^ reduction was monitored by an increase of the absorbance at 534 nm due to complex formation with the Fe^2+^-specific chelator bathophenanthroline disulfonic acid (BPS) (76, 77) in 96-well microtiter plates containing 100 μl of sample. The iron reduction assay was performed in 200 mM MOPS buffer (pH 7.4), 0.5 mM FeCl_3_ was added from a stock in 10 mM HCl neutralized with 50 mM NaOH immediately before use. 19.5 μM PCA, other benzoic acid derivatives, or 50 mM NaHCO_3_ was applied as indicated. The reaction was started by the addition of 6.5 mM BPS and measured every 10 min.

### Fe^3+^-PCA complex formation.

Complex formation between Fe^3+^ and PCA was monitored in a setup identical to the setup used in the iron reduction assay but in the absence of Fe^2+^-specific BPS. The Fe^3+^-PCA complex formation assay was started by the addition of 1.5 mM PCA to provide it in a 3:1 stoichiometry with iron. Fe^3+^-catecholate complexes show characteristic λ_max_ values between 561 and 586 nm (20), and for Fe^3+^-PCA, 575 nm was reported as λ_max_ (19). Wavelength scans of the complexes were performed with 100-μl samples in 96-well plates (1-nm step size) after 5 h of incubation. Since the absorbance peaked around 560 nm in the standard setup (see Fig. S5 in the supplemental material), kinetic measurements of complex formation was performed in the following experiments every 5 min at 560 nm in the same setup.

10.1128/mBio.00085-20.5 FIG S5 Wavelength scan of the Fe^3+^-PCA complex between 400 and 700 nm (1-nm step width). The solid line represents the mean values with the error bars indicating the standard deviations of three independent experiments. Download FIG S5, EPS file, 0.2 MB. Copyright © 2020 Müller et al. 2020 Müller et al. This content is distributed under the terms of the Creative Commons Attribution 4.0 International license.

### Purification of DtxR.

For the production of DtxR protein, a cryogenic culture of E. coli DH5α(pJOE6089-*dtxR*) was streaked on a 2× YT agar plate containing 100 μg ampicillin (Amp^100^) ml^−1^ and incubated for 24 h at 37°C. A single cell was used to inoculate 5 ml of 2× YT (Amp^100^) which was incubated overnight (O/N) at 37°C on a rotary shaker at 120 rpm before 1.5 ml of this culture was transferred to fresh 150 ml of 2× YT (Amp^100^) medium in a 1-liter baffled shaking flask. This culture was incubated at 37°C on a rotary shaker at 120 rpm until an OD_600_ of 0.84 was reached. The expression of *dtxR* was induced by the addition of l-rhamnose to a final concentration of 2 g liter^−1^, and incubation continued for 6 h at 30°C before the cells were harvested by centrifugation (10 min, 5,000 rpm at 4°C in an Eppendorf centrifuge 5804 R). The cell pellets were stored frozen at –20°C until further processing.

Strep-tagged protein was purified with the Strep-tagged protein purification kit (Qiagen, Hilden, Germany) after lysing cells by sonication (five cycles, Sonopuls HD2200, Bandelin, Berlin, Germany; tip type MS73, 30 s, power 40%).

### Thermal shift assay.

Differential scanning fluorimetry experiments were performed in a final sample volume of 50 μl in 96-well plates using the Mastercycler EP Realplex^2^ (epgradient S; Eppendorf, Hamburg, Germany). Briefly, at a gradually increasing temperature, protein denaturation is monitored by an increase of the fluorescence which is caused by the binding of a dye to successively presented hydrophobic areas of the protein (78). Ligand binding might increase the protein stability. Thermal protein denaturation was monitored by an increase of the absorbance at 550 nm due to binding of SYPRO Orange (Thermo Fisher Scientific Inc., Bremen, Germany).

Protein samples (50 μg ml^−1^) were prepared in 100 mM potassium phosphate buffer (pH 7.4) containing 600 mM NaCl and 5 mM dithiothreitol (DTT). Divalent metal ions were applied at various concentrations (0.1 μM to 1 mM NiCl_2_ or 0.1 μM to 5 mM MnCl_2_). When 10 to 200 mM NaHCO_3_ was added to the sample, osmolarity was maintained at an identical level by adjusting the NaCl concentration. One microliter of SYPRO Orange was added to each sample immediately before starting the assay. Then, the 96-well microtiter plate was sealed with adhesive foil and incubated in the mastercycler (20°C for 15 s, 20°C to 90°C in 30 min, 90°C for 15 s). The range of the greatest absorbance increase over temperature was fitted by a fourth order polynomial (best fit), and the melting temperature (*T_m_*) was determined at the inflection point. The *T_m_* at a given ligand concentration eventually represents the mean value ± standard deviation for at least four replicates. Dissociation constants (*K_D_*) were calculated for the inflection point of the *T_m_* on the ligand concentration curve (79).

### Statistical analysis.

All growth experiments were performed as independent biological triplicates on different days. (Bio)chemical assays were replicated 4 to 6 times, and LC-MS-QTOF analysis was performed on the three biological replicate samples. All values represent mean values with error bars indicating standard deviations. When appropriate, statistically significant differences of sample means were tested with a two-sample *t* test.

## References

[1] Proulx-Curry PM, Chasteen ND 1995 Molecular aspects of iron uptake and storage in ferritin. Coord Chem Rev 144:347–368. doi:10.1016/0010-8545(95)01148-I.

[2] Andrews SC, Robinson AK, Rodríguez-Quiñones F 2003 Bacterial iron homeostasis. FEMS Microbiol Rev 27:215–237. doi:10.1016/S0168-6445(03)00055-X. 12829269

[3] Neilands JB 1981 Microbial iron compounds. Annu Rev Biochem 50:715–731. doi:10.1146/annurev.bi.50.070181.003435. 6455965

[4] Schröder I, Johnson E, De Vries S 2003 Microbial ferric iron reductases. FEMS Microbiol Rev 27:427–447. doi:10.1016/S0168-6445(03)00043-3. 12829278

[5] Light SH, Su L, Rivera-Lugo R, Cornejo JA, Louie A, Iavarone AT, Ajo-Franklin CM, Portnoy DA 2018 A flavin-based extracellular electron transfer mechanism in diverse Gram-positive bacteria. Nature 562:140–157. doi:10.1038/s41586-018-0498-z. 30209391 PMC6221200

[6] Kalinowski J, Bathe B, Bartels D, Bischoff N, Bott M, Burkovski A, Dusch N, Eggeling L, Eikmanns BJ, Gaigalat L, Goesmann A, Hartmann M, Huthmacher K, Krämer R, Linke B, McHardy AC, Meyer F, Möckel B, Pfefferle W, Pühler A, Rey DA, Rückert C, Rupp O, Sahm H, Wendisch VF, Wiegräbe I, Tauch A 2003 The complete *Corynebacterium glutamicum* ATCC 13032 genome sequence and its impact on the production of l-aspartate-derived amino acids and vitamins. J Biotechnol 104:5–25. doi:10.1016/s0168-1656(03)00154-8. 12948626

[7] Frunzke J, Bott M 2008 Regulation of iron homeostasis in *Corynebacterium glutamicum*, p 241–266. *In* Burkovski A (ed), Corynebacteria: genomics and molecular biology. Horizon Scientific Press, Norwich, United Kingdom.

[8] von der Osten CH, Gioannetti C, Sinskey AJ 1989 Design of a defined medium for growth of *Corynebacterium glutamicum* in which citrate facilitates iron uptake. Biotechnol Lett 11:11–16. doi:10.1007/BF01026778.

[9] Liebl W, Klamer R, Schleifer K-H 1989 Requirement of chelating compounds for the growth of *Corynebacterium glutamicum* in synthetic media. Appl Microbiol Biotechnol 32:205–210. doi:10.1007/BF00165889.

[10] Keilhauer C, Eggeling L, Sahm H 1993 Isoleucine synthesis in *Corynebacterium glutamicum*: molecular analysis of the *ilvB-ilvN-ilvC* operon. J Bacteriol 175:5595–5603. doi:10.1128/jb.175.17.5595-5603.1993. 8366043 PMC206616

[11] Küberl A, Polen T, Bott M 2016 The pupylation machinery is involved in iron homeostasis by targeting the iron storage protein ferritin. Proc Natl Acad Sci U S A 113:4806–4811. doi:10.1073/pnas.1514529113. 27078093 PMC4855571

[12] Peters WJ, Warren RA 1968 Itoic acid synthesis in *Bacillus subtilis*. J Bacteriol 95:360–366. doi:10.1128/JB.95.2.360-366.1968. 4966543 PMC252027

[13] Tait GH 1975 The identification and biosynthesis of siderochromes formed by *Micrococcus denitrificans*. Biochem J 146:191–204. doi:10.1042/bj1460191. 238503 PMC1165288

[14] Peters WJ, Warren R 1968 Phenolic acids and iron transport in *Bacillus subtilis*. Biochim Biophys Acta 165:225–232. doi:10.1016/0304-4165(68)90050-0. 4971642

[15] Barbeau K, Zhang G, Live DH, Butler A 2002 Petrobactin, a photoreactive siderophore produced by the oil-degrading marine bacterium *Marinobacter hydrocarbonoclasticus*. J Am Chem Soc 124:378–379. doi:10.1021/ja0119088. 11792199

[16] Garner BL, Arceneaux JEL, Byers BR 2004 Temperature control of a 3,4-dihydroxybenzoate (protocatechuate)-based siderophore in *Bacillus anthracis*. Curr Microbiol 49:89–94. doi:10.1007/s00284-004-4286-7. 15297912

[17] Ratledge C, Chaudhry MA 1971 Accumulation of iron-binding phenolic acids by Actinomycetales and other organisms related to the Mycobacteria. J Gen Microbiol 66:71–78. doi:10.1099/00221287-66-1-71. 5559616

[18] Zawadzka AM, Abergel RJ, Nichiporuk R, Andersen UN, Raymond KN 2009 Siderophore-mediated iron acquisition systems in *Bacillus cereus*: identification of receptors for anthrax virulence-associated petrobactin. Biochemistry 48:3645–3657. doi:10.1021/bi8018674. 19254027 PMC2782674

[19] Kennedy JA, Powell J 1985 Aluminium(III) and iron(III) 1,2-diphenolato complexes: a potentiometric study. Aust J Chem 38:659–667. doi:10.1071/CH9850659.

[20] Perron NR, Brumaghim JL 2009 A review of the antioxidant mechanisms of polyphenol compounds related to iron binding. Cell Biochem Biophys 53:75–100. doi:10.1007/s12013-009-9043-x. 19184542

[21] Avdeef A, Sofen SR, Bregante TL, Raymond KN 1978 Coordination chemistry of microbial iron transport compounds. 9. Stability constants for catechol models of enterobactin. J Am Chem Soc 100:5362–5370. doi:10.1021/ja00485a018.

[22] Blombach B, Takors R 2015 CO_2_ − intrinsic product, essential substrate, and regulatory trigger of microbial and mammalian production processes. Front Bioeng Biotechnol 3:108. doi:10.3389/fbioe.2015.00108. 26284242 PMC4522908

[23] Cummins EP, Selfridge AC, Sporn PH, Sznajder JI, Taylor CT 2014 Carbon dioxide-sensing in organisms and its implications for human disease. Cell Mol Life Sci 71:831–845. doi:10.1007/s00018-013-1470-6. 24045706 PMC3945669

[24] Lopes M, Belo I, Mota M 2014 Over-pressurized bioreactors: application to microbial cell cultures. Biotechnol Prog 30:767–775. doi:10.1002/btpr.1917. 24777971

[25] Yu T, Chen Y 2019 Effects of elevated carbon dioxide on environmental microbes and its mechanisms: a review. Sci Total Environ 655:865–879. doi:10.1016/j.scitotenv.2018.11.301. 30481713

[26] Ueda K, Tagami Y, Kamihara Y, Shiratori H, Takano H, Beppu T 2008 Isolation of bacteria whose growth is dependent on high levels of CO_2_ and implications of their potential diversity. Appl Environ Microbiol 74:4535–4538. doi:10.1128/AEM.00491-08. 18487395 PMC2493168

[27] Sauer U, Eikmanns BJ 2005 The PEP-pyruvate-oxaloacetate node as the switch point for carbon flux distribution in bacteria. FEMS Microbiol Rev 29:765–794. doi:10.1016/j.femsre.2004.11.002. 16102602

[28] Inui M, Murakami S, Okino S, Kawaguchi H, Vertès AA, Yukawa H 2004 Metabolic analysis of *Corynebacterium glutamicum* during lactate and succinate productions under oxygen deprivation conditions. J Mol Microbiol Biotechnol 7:182–196. doi:10.1159/000079827. 15383716

[29] Okino S, Noburyu R, Suda M, Jojima T, Inui M, Yukawa H 2008 An efficient succinic acid production process in a metabolically engineered *Corynebacterium glutamicum* strain. Appl Microbiol Biotechnol 81:459–464. doi:10.1007/s00253-008-1668-y. 18777022

[30] Blombach B, Buchholz J, Busche T, Kalinowski J, Takors R 2013 Impact of different CO_2_/HCO_3_^−^ levels on metabolism and regulation in *Corynebacterium glutamicum*. J Biotechnol 168:331–340. doi:10.1016/j.jbiotec.2013.10.005. 24140290

[31] Michel A, Koch-Koerfges A, Krumbach K, Brocker M, Bott M 2015 Anaerobic growth of *Corynebacterium glutamicum* via mixed-acid fermentation. Appl Environ Microbiol 81:7496–7508. doi:10.1128/AEM.02413-15. 26276118 PMC4592858

[32] Wennerhold J, Bott M 2006 The DtxR regulon of *Corynebacterium glutamicum*. J Bacteriol 188:2907–2918. doi:10.1128/JB.188.8.2907-2918.2006. 16585752 PMC1446976

[33] Litwin CM, Calderwood SB 1993 Role of iron in regulation of virulence genes. Clin Microbiol Rev 6:137–149. doi:10.1128/cmr.6.2.137. 8472246 PMC358274

[34] Mekalanos JJ 1992 Environmental signals controlling expression of virulence determinants in bacteria. J Bacteriol 174:1–7. doi:10.1128/jb.174.1.1-7.1992. 1729202 PMC205668

[35] Wilson BR, Bogdan AR, Miyazawa M, Hashimoto K, Tsuji Y 2016 Siderophores in iron metabolism: from mechanism to therapy potential. Trends Mol Med 22:1077–1090. doi:10.1016/j.molmed.2016.10.005. 27825668 PMC5135587

[36] D’Aquino JA, Denninger AR, Moulin AG, D’Aquino KE, Ringe D 2009 Decreased sensitivity to changes in the concentration of metal ions as the basis for the hyperactivity of DtxR(E175K). J Mol Biol 390:112–123. doi:10.1016/j.jmb.2009.05.003. 19433095

[37] Qiu X, Verlinde CL, Zhang S, Schmitt MP, Holmes RK, Hol WG 1995 Three-dimensional structure of the diphtheria toxin repressor in complex with divalent cation co-repressors. Structure 3:87–100. doi:10.1016/s0969-2126(01)00137-x. 7743135

[38] Qiu X, Pohl E, Holmes RK, Hol W 1996 High-resolution structure of the diphtheria toxin repressor complexed with cobalt and manganese reveals an SH3-like third domain and suggests a possible role of phosphate as co-corepressor. Biochemistry 35:12292–12302. doi:10.1021/bi960861d. 8823163

[39] Goranson-Siekierke J, Pohl E, Hol WGJ, Holmes RK 1999 Anion-coordinating residues at binding site 1 are essential for the biological activity of the diphtheria toxin repressor. Infect Immun 67:1806–1811. 10085021 10.1128/iai.67.4.1806-1811.1999 PMC96531

[40] Unthan S, Grünberger A, van Ooyen J, Gätgens J, Heinrich J, Paczia N, Wiechert W, Kohlheyer D, Noack S 2014 Beyond growth rate 0.6: what drives *Corynebacterium glutamicum* to higher growth rates in defined medium. Biotechnol Bioeng 111:359–371. doi:10.1002/bit.25103. 23996851

[41] Grünberger A, van Ooyen J, Paczia N, Rohe P, Schiendzielorz G, Eggeling L, Wiechert W, Kohlheyer D, Noack S 2013 Beyond growth rate 0.6: *Corynebacterium glutamicum* cultivated in highly diluted environments. Biotechnol Bioeng 110:220–228. doi:10.1002/bit.24616. 22890752

[42] Frunzke J, Gätgens C, Brocker M, Bott M 2011 Control of heme homeostasis in *Corynebacterium glutamicum* by the two-component system HrrSA. J Bacteriol 193:1212–1221. doi:10.1128/JB.01130-10. 21217007 PMC3067591

[43] Davison W, Seed G 1983 The kinetics of the oxidation of ferrous iron in synthetic and natural waters. Geochim Cosmochim Acta 47:67–79. doi:10.1016/0016-7037(83)90091-1.

[44] Morgan LR 1962 Oxidation of protocatechuic acid with peroxyacetic acid. J Org Chem 27:1208–1210. doi:10.1021/jo01051a020.

[45] Shen XH, Zhou NY, Liu SJ 2012 Degradation and assimilation of aromatic compounds by *Corynebacterium glutamicum*: another potential for applications for this bacterium? Appl Microbiol Biotechnol 95:77–89. doi:10.1007/s00253-012-4139-4. 22588501

[46] Boyer RF, Clark HM, LaRoche AP 1988 Reduction and release of ferritin iron by plant phenolics. J Inorg Biochem 32:171–181. doi:10.1016/0162-0134(88)80025-4. 3131480

[47] Nyhus KJ, Wilborn AT, Jacobson ES 1997 Ferric iron reduction by *Cryptococcus neoformans*. Infect Immun 65:434–438. doi:10.1128/IAI.65.2.434-438.1997. 9009293 PMC174613

[48] Teramoto H, Inui M, Yukawa H 2009 Regulation of expression of genes involved in quinate and shikimate utilization in *Corynebacterium glutamicum*. Appl Environ Microbiol 75:3461–3468. doi:10.1128/AEM.00163-09. 19376919 PMC2687277

[49] Kubota T, Tanaka Y, Takemoto N, Watanabe A, Hiraga K, Inui M, Yukawa H 2014 Chorismate-dependent transcriptional regulation of quinate/shikimate utilization genes by LysR-type transcriptional regulator QsuR in *Corynebacterium glutamicum*: carbon flow control at metabolic branch point. Mol Microbiol 92:356–368. doi:10.1111/mmi.12560. 24674055

[50] Schweigert N, Zehnder AJB, Eggen R 2001 Chemical properties of catechols and their molecular modes of toxic action in cells, from microorganisms to mammals. Environ Microbiol 3:81–91. doi:10.1046/j.1462-2920.2001.00176.x. 11321547

[51] Ding H, Clark RJ 2004 Characterization of iron binding in IscA, an ancient iron-sulphur cluster assembly protein. Biochem J 379:433–440. doi:10.1042/BJ20031702. 14720122 PMC1224081

[52] Ding H, Harrison K, Lu J 2005 Thioredoxin reductase system mediates iron binding in IscA and iron delivery for the iron-sulfur cluster assembly in IscU. J Biol Chem 280:30432–30437. doi:10.1074/jbc.M504638200. 15985427

[53] Aisen P, Leibman A, Zweier J 1978 Stoichiometric and site characteristics of the binding of iron to human transferrin. J Biol Chem 253:1930–1937. 204636

[54] Burton CL, Chhabra SR, Swift S, Baldwin TJ, Withers H, Hill SJ, Williams P 2002 The growth response of *Escherichia coli* to neurotransmitters and related catecholamine drugs requires a functional enterobactin biosynthesis and uptake system. Infect Immun 70:5913–5923. doi:10.1128/iai.70.11.5913-5923.2002. 12379665 PMC130287

[55] Freestone PPE, Haigh RD, Williams PH, Lyte M 2003 Involvement of enterobactin in norepinephrine-mediated iron supply from transferrin to enterohaemorrhagic *Escherichia coli*. FEMS Microbiol Lett 222:39–43. doi:10.1016/S0378-1097(03)00243-X. 12757944

[56] Anderson MT, Armstrong SK 2008 Norepinephrine mediates acquisition of transferrin-iron in *Bordetella bronchiseptica*. J Bacteriol 190:3940–3947. doi:10.1128/JB.00086-08. 18390651 PMC2395024

[57] Coulanges V, Andre P, Vidon D 1998 Effect of siderophores, catecholamines, and catechol compounds on *Listeria* spp. growth in iron-complexed medium. Biochem Biophys Res Commun 249:526–530. doi:10.1006/bbrc.1998.9184. 9712730

[58] Lyte M, Ernst S 1992 Catecholamine induced growth of Gram negative bacteria. Life Sci 50:203–212. doi:10.1016/0024-3205(92)90273-r. 1731173

[59] Freestone PPE, Lyte M, Neal CP, Maggs AF, Haigh RD, Williams PH 2000 The mammalian neuroendocrine hormone norepinephrine supplies iron for bacterial growth in the presence of transferrin or lactoferrin. J Bacteriol 182:6091–6098. doi:10.1128/jb.182.21.6091-6098.2000. 11029429 PMC94743

[60] Freestone PPE, Walton NJ, Haigh RD, Lyte M 2007 Influence of dietary catechols on the growth of enteropathogenic bacteria. Int J Food Microbiol 119:159–169. doi:10.1016/j.ijfoodmicro.2007.07.039. 17850907

[61] Sandrini SM, Shergill R, Woodward J, Muralikuttan R, Haigh RD, Lyte M, Freestone PP 2010 Elucidation of the mechanism by which catecholamine stress hormones liberate iron from the innate immune defense proteins transferrin and lactoferrin. J Bacteriol 192:587–594. doi:10.1128/JB.01028-09. 19820086 PMC2805316

[62] Torres VJ, Attia AS, Mason WJ, Hood MI, Corbin BD, Beasley FC, Anderson KL, Stauff DL, McDonald WH, Zimmerman LJ, Friedman DB, Heinrichs DE, Dunman PM, Skaar EP 2010 *Staphylococcus aureus* Fur regulates the expression of virulence factors that contribute to the pathogenesis of pneumonia. Infect Immun 78:1618–1628. doi:10.1128/IAI.01423-09. 20100857 PMC2849423

[63] Lin CT, Wu CC, Chen YS, Lai YC, Chi C, Lin JC, Chen Y, Peng HL 2011 Fur regulation of the capsular polysaccharide biosynthesis and iron-acquisition systems in *Klebsiella pneumoniae* CG43. Microbiology 157:419–429. doi:10.1099/mic.0.044065-0. 21071493

[64] Zondervan NA, Van Dam JCJ, Schaap PJ, Martins Dos Santos VAP, Suarez-Diez M 2018 Regulation of three virulence strategies of *Mycobacterium tuberculosis*: a success story. Int J Mol Sci 19:E347. doi:10.3390/ijms19020347. 29364195 PMC5855569

[65] Sambrook J, Russell RW 2001 Molecular cloning: a laboratory manual, 3rd ed. Cold Spring Harbor Laboratory Press, Cold Spring Harbor, NY.

[66] Lange J, Müller F, Takors R, Blombach B 2018 Harnessing novel chromosomal integration loci to utilize an organosolv-derived hemicellulose fraction for isobutanol production with engineered *Corynebacterium glutamicum*. Microb Biotechnol 11:257–263. doi:10.1111/1751-7915.12879. 29115043 PMC5743825

[67] Hoffmann J, Altenbuchner J 2014 Hyaluronic acid production with *Corynebacterium glutamicum*: effect of media composition on yield and molecular weight. J Appl Microbiol 117:663–678. doi:10.1111/jam.12553. 24863652

[68] Schäfer A, Tauch A, Jäger W, Kalinowski J, Thierbach G, Pühler A 1994 Small mobilizable multi-purpose cloning vectors derived from the *Escherichia coli* plasmids pK18 and pK19: selection of defined deletions in the chromosome of *Corynebacterium glutamicum*. Gene 145:69–73. doi:10.1016/0378-1119(94)90324-7. 8045426

[69] Gibson DG 2011 Enzymatic assembly of overlapping DNA fragments. Methods Enzymol 498:349–361. doi:10.1016/B978-0-12-385120-8.00015-2. 21601685 PMC7149801

[70] Cordes C, Möckel B, Eggeling L, Sahm H 1992 Cloning, organization and functional analysis of *ilvA*, *ilvB* and *ilvC* genes from *Corynebacterium glutamicum*. Gene 112:113–116. doi:10.1016/0378-1119(92)90311-c. 1551588

[71] Eikmanns BJ, Metzger M, Reinscheid D, Kircher M, Sahm H 1991 Amplification of three threonine biosynthesis genes in *Corynebacterium glutamicum* and its influence on carbon flux in different strains. Appl Microbiol Biotechnol 34:617–622. doi:10.1007/bf00167910. 1369320

[72] Schwentner A, Feith A, Münch E, Stiefelmaier J, Lauer I, Favilli L, Massner C, Öhrlein J, Grund B, Hüser A, Takors R, Blombach B 2019 Modular systems metabolic engineering enables balancing of relevant pathways for l-histidine production with *Corynebacterium glutamicum*. Biotechnol Biofuels 12:1–21. doi:10.1186/s13068-019-1410-2. 30962820 PMC6432763

[73] Failmezger J, Nitschel R, Sánchez-Kopper A, Kraml M, Siemann-Herzberg M 2016 Site-specific cleavage of ribosomal RNA in *Escherichia coli*-based cell-free protein synthesis systems. PLoS One 11:e0168764. doi:10.1371/journal.pone.0168764. 27992588 PMC5167549

[74] Teleki A, Sánchez-Kopper A, Takors R 2015 Alkaline conditions in hydrophilic interaction liquid chromatography for intracellular metabolite quantification using tandem mass spectrometry. Anal Biochem 475:4–13. doi:10.1016/j.ab.2015.01.002. 25600449

[75] Feith A, Teleki A, Graf M, Favilli L, Takors R 2019 HILIC-enabled ^13^C metabolomics strategies: comparing quantitative precision and spectral accuracy of QTOF high- and QQQ low-resolution mass spectrometry. Metabolites 9:E63. doi:10.3390/metabo9040063. 30986989 PMC6523712

[76] Cowart RE, Singleton FL, Hind JS 1993 A comparison of bathophenanthrolinedisulfonic acid and ferrozine as chelators of iron(II) in reduction reactions. Anal Biochem 211:151–155. doi:10.1006/abio.1993.1246. 8323027

[77] Blair D, Diehl H 1961 Bathophenanthrolinedisulphonic acid and bathocuproinedisulphonic acid, water soluble reagents for iron and copper. Talanta 7:163–174. doi:10.1016/0039-9140(61)80006-4.

[78] Niesen FH, Berglund H, Vedadi M 2007 The use of differential scanning fluorimetry to detect ligand interactions that promote protein stability. Nat Protoc 2:2212–2221. doi:10.1038/nprot.2007.321. 17853878

[79] Vivoli M, Novak HR, Littlechild JA, Harmer NJ 2014 Determination of protein-ligand interactions using differential scanning fluorimetry. J Vis Exp 2014:51809. doi:10.3791/51809. PMC4692391 25285605

