# Supplementary material for: CO2/HCO3− Accelerates Iron Reduction through Phenolic Compounds
Source: mBio. 2020 Mar 10;11(2):e00085-20. doi: 10.1128/mBio.00085-20 (PMC7064749; doi:10.1128/mBio.00085-20)
Supplement: TABLE S1 [file mBio.00085-20-st001.docx]

Table S1: Oligonucleotides used for PCR amplification and sequencing; overlaps with neighbouring sequences (Gibson Assembly) are shown in blue, restriction sites are underlined.

| **Name** | **Sequence (5‘ → 3‘)** | **Purpose** |
| --- | --- | --- |
| ftn1 | AACAGCTATGACCATGATTACGCCAAGCTTAGCATCGTGAACACTGAG | Forward primer 5‘flank in pK19*mobsacB*-∆*ftn* |
| ftn2 | AACTGCGGGGAATGGCCCCTAACGTACACAAC | reverse primer 5‘flank in pK19*mobsacB*-∆*ftn* |
| ftn3 | TACGTTAGGGGCCATTCCCCGCAGTTTTTAATG | Forward primer 3‘flank in pK19*mobsacB*-∆*ftn* |
| ftn4 | AGTGAATTCGAGCTCGGTACCCGGGGATCCCAGCGAATGATGGACTTATC | reverse primer 3‘flank in pK19*mobsacB*-∆*ftn* |
| dps1 | AACAGCTATGACCATGATTACGCCAAGCTTTGCGCTCTGCATCGGTGA | Forward primer 5‘flank in pK19*mobsacB*-∆*dps* |
| dps2 | GCTTTTCGACGTTGACCCCTATTATAGCACTGCTTGATAAATTG | reverse primer 5‘flank in pK19*mobsacB*-∆*dps* |
| dps3 | TATAATAGGGGTCAACGTCGAAAAGCGTTAAGGCGC | Forward primer 3‘flank in pK19*mobsacB*-∆*dps* |
| dps4 | AGTGAATTCGAGCTCGGTACCCGGGGATCCGGCAAGGCCCCAGGGAGG | reverse primer 3‘flank in pK19*mobsacB*-∆*dps* |
| 5‘(cg3344)-1 | CAGGTCGACTCTAGAGGATCCGTTCCGAAGAAGCCAAAG | forward primer 5’flank in pK19*mobsacB*-P_ripA_-*lacI* |
| 5‘(cg3344)-2 | CTTTGCGAGCAGGTCGCTGCGAAATGGAAAACGCCCC | reverse primer 5’flank in pK19*mobsacB*-P_ripA_-*lacI* |
| Pripa-1 | CCGGGGCGTTTTCCATTTCGCAGCGACCTGC | forward primer P_ripA_ in pK19*mobsacB*-P_ripA_-*lacI* |
| Pripa-2 | ATCTCATCCTCACTACAAGC | reverse primer P_ripA_ in pK19*mobsacB*-P_ripA_-*lacI* |
| lacI-1 | GCTTGTAGTGAGGATGAGATGTGGTGAATGTGAAACCAG | forward primer *lacI* in pK19*mobsacB*-P_ripA_-*lacI* |
| lacI-2 | CATCCGCCAAAACAGCCAAGCTCACTGCCCGCTTTC | reverse primer *lacI* in pK19*mobsacB*-P_ripA_-*lacI* |
| TrrnB-1 | GCTTGGCTGTTTTGGC | forward primer T_rrnB_ in pK19*mobsacB*-P_ripA_-*lacI* |
| TrrnB-2 | CAGGAGAGCGTTCACC | reverse primer T_rrnB_ in pK19*mobsacB*-P_ripA_-*lacI* |
| 3‘(cg3345)-1 | GTCGGTGAACGCTCTCCTGACACATCTGTTCGACTCGC | forward primer 3’flank in pK19*mobsacB*-P_ripA_-*lacI* |
| 3’(cg3345)-2 | GCGGCAGCGTGAAGCTAGCCGCGTGATTCCTGGAAAATTAG | reverse primer 3’flank in pK19*mobsacB*-P_ripA_-*lacI* |
| Ptac-1 | GCGACGCCGCAGGGTCTAGACCCTGAATTGACTCTCTTCC | Forward primer P_tac_ in pJC4-P_tac_-*egfp* |
| Ptac-2 | ATGGTATATCTCCTTCAATTCTG | reverse primer P_tac_ in pJC4-P_tac_-*egfp* |
| egfp-1 | GAATTGAAGGAGATATACCATATGGTGAGCAAGGGCGAGGAG | Forward primer *egfp*-T_rrnB_ in pJC4-P_tac_-*egfp* |
| egfp-2 | GATATCCATCACACTGGCGGCCGCCAGGAGAGCGTTCACC | reverse primer *egfp*-T_rrnB_ in pJC4-P_tac_-*egfp* |
| dtxR-1 | GGGAATTCCATATGAAGGATCTGGTCGATACCACCG | Forward primer for *dtxR* in pJOE6089-*dtxR* |
| dtxR-2 | CGCGGATCCGCCCTCAACCTTTTCTACGCGG | reverse primer for *dtxR* in pJOE6089-*dtxR* |
| **Sequencing Primer** |  |  |
| fw_pJC4_seq | CGATTGAAGACCGTCAAC | Generic forward primer for sequencing of pJC4 based plasmids |
| rev_pJC4_seq | GTCATCAGACCAAGGAG | Generic reverse primer for sequencing of pJC4 based plasmids |
| fw_pK19_seq | CAGGCTTTACACTTTATGC | Generic forward primer for sequencing of pK19*mobsacB* based plasmids |
| rev_pK19_seq | ACCTGCTTTCTCTTTGCG | Generic reverse primer for sequencing of pK19*mobsacB* based plasmids |
| fw_Ptac_seq | AGCCATCGGAAGCTGTG | Forward sequencing primer binding inside P_tac_ |
| fw_PripA_seq | GAGATCCCAGAGGCATAG | Forward sequencing primer binding inside P_ripA_ |
| fw_egfp_seq | ACAACATCGAGGACGGC | Forward sequencing primer binding inside *egfp* |
| rev_egfp_seq | TCAGCTTGCCGTAGGTG | reverse sequencing primer binding inside *egfp* |
| fw_lacI_seq | AGCAAATCGCGCTGTTAG | Forward sequencing primer binding inside *lacI* |
| rev_lacI_seq | GTTGAAAACCGGACATGG | reverse sequencing primer binding inside *lacI* |
| rev_TrrnB_seq | TACGGCGTTTCACTTCTG | reverse sequencing primer binding inside T_rrnB_ |
| fw_5'ftn_seq | TTAGACCTAGCTTTAGAGC | Forward sequencing primer for *ftn* deletion construct |
| rev_3'ftn_seq | TGTGGCAAAACAGTAATGC | reverse sequencing primer for *ftn* deletion construct |
| fw_5'dps_seq | GTGACCGTTGATGATCGC | Forward sequencing primer for *dps* deletion construct |
| rev_3'dps_seq | CTTAGAGGCGATTCTAGG | reverse sequencing primer for *dps* deletion construct |
