# Supplementary material for: CO2/HCO3− Accelerates Iron Reduction through Phenolic Compounds
Source: mBio. 2020 Mar 10;11(2):e00085-20. doi: 10.1128/mBio.00085-20 (PMC7064749; doi:10.1128/mBio.00085-20)
Supplement: TABLE S2 [file mBio.00085-20-st002.docx]

Table S2: Overview of the strains and plasmids used in this study.

| **Strain or Plasmid** | **Relevant Characteristics** | **Reference / Source** |
| --- | --- | --- |
| *E. coli* DH5α | *supE44* ∆*lacU169 (f80lacZ*∆*M15) hsdR17 recA1 endA1 gyrA96 thi relA1* | (7) |
| *C. glutamicum* ATCC 13032 | wildtype strain (WT) | (8), American Type Culture Collection |
| *E. coli* DH5α (pJOE6089-*dtxR*) | rhamnose inducible *dtxR* overexpression, C-terminal Strep tag II | this work |
| *C. glutamicum* FEM3 | *C. glutamicum* ATCC 13032 (*cg3344-cg3345*)’::P_ripA_-*lacI* (pJC4-P_tac_-*egfp* ) | this work |
| *C. glutamicum* ATCC 13032 ∆*pup* | deletion of the *pup* gene (*cg1689*) in *C. glutamicum* WT using pK19*mobsacB*-∆*pup* | this work |
| *C. glutamicum* ATCC 13032 ∆*ftn* | deletion of the *ftn* gene (*cg2782*) in *C. glutamicum* WT | this work |
| *C. glutamicum* ATCC 13032 ∆*dps* | deletion of the *dps* gene (*cg3327*) in *C. glutamicum* WT | this work |
| *C. glutamicum* ATCC 13032 ∆*ftn* ∆*dps* | double deletion of the *ftn* (*cg2782*) and *dps* (*cg3327*) genes in *C. glutamicum* WT | this work |
| *C. glutamicum* FEM3 ∆*pup* | deletion of the *pup* gene (*cg1689*) in *C. glutamicum* FEM3 using pK19*mobsacB*-∆*pup* | this work |
| *C. glutamicum* FEM3 ∆*ftn* | deletion of the *ftn* gene (*cg2782*) in *C. glutamicum* FEM3 | this work |
| *C. glutamicum* FEM3 ∆*dps* | deletion of the *dps* gene (*cg3327*) in *C. glutamicum* FEM3 | this work |
| *C. glutamicum* FEM3 ∆*ftn* ∆*dps* | double deletion of the *ftn* (*cg2782*) and *dps* (*cg3327*) genes in *C. glutamicum* FEM3 | this work |
| **Plasmids:** |  |  |
|  |  |  |
| pJOE7706.1 | IPTG inducible overexpression shuttle plasmid for *E. coli* and *C. glutamicum*; pBR322 oriV_E. coli_, pCG1 oriV_C. glutamicum_, *lacI*^q^-P_tac_-*egfp*-T_rrnB_, kan^R^ | (3) |
| pJOE6089.4 | rhamnose inducible overexpression plasmid in *E. coli*; pBR322 oriV_E. coli_, P_rhaBAD_, *egfp*-*Strep*-tag II (C-terminal), T_rrnB_, amp^R^ | (9) |
| pJOE6089-*dtxR* | rhamnose inducible overexpression plasmid in *E. coli*; pBR322 oriV_E. coli_, P_rhaBAD_, *dtxR*-*Strep*-tag II (C-terminal), T_rrnB_, amp^R^ | This work |
| pK19*mobsacB* | shuttle vector for amplification in *E. coli* and integration/deletion in *C. glutamicum*; pMB1 oriV_E. coli_, oriT (RP4*mob*), *lacZ*α, *sacB*_B. subtilis_, Kan^R^ | (4) |
| pJC4 | *E. coli*-*C. glutamicum* shuttle vector; oriV_E. coli_, pCG1 oriV_C. glutamicum_, Kan^R^ | (6) |
| pK19*mobsacB*- flank1(3’*cg3344*)- P_ripA_-*lacI*-T_rrnB_- flank2(5’*cg3345*) | plasmid for the integration of *lacI* under control of the *ripA* promoter (P_ripA_) in the intergenic region between *cg3344* and *cg3345* (defined as CgLP13 by Lange *et al.* (2017)) in the *C. glutamicum* genome; all fragments inserted simultaneously in BamHI and NheI linearized pK19*mobsacB* | this work |
| pJC4-P_tac_-*egfp* | P_tac_ -*egfp*-T_rrnB_ inserted in XbaI and NotI linearized pJC4 for controlled expression of *egfp* (in FEM3 and derivatives) | this work |
| pK19*mobsacB*-∆*pup* | pK19*mobsacB* derivative for markerless deletion of the *pup* gene (*cg1689*) in *C. glutamicum* | (10) |
| pK19*mobsacB*-∆*ftn* | pK19*mobsacB* derivative for markerless deletion of the *ftn* gene (*cg2782*) in *C. glutamicum*; homologous sequences* inserted in HindIII and BamHI linearized pK19*mobsacB* plasmid backbone | this work |
| pK19*mobsacB*-∆*dps* | pK19*mobsacB* derivative for markerless deletion of the *dps* gene (*cg3327*) in *C. glutamicum*; homologous sequences* inserted in HindIII and BamHI linearized pK19*mobsacB* plasmid backbone | this work |
